# The P2X7 Receptor 489C>T Gain of Function Polymorphism Favors HHV-6A Infection and Associates With Female Idiopathic Infertility

**DOI:** 10.3389/fphar.2020.00096

**Published:** 2020-02-21

**Authors:** Anna Pegoraro, Daria Bortolotti, Roberto Marci, Elisabetta Caselli, Simonetta Falzoni, Elena De Marchi, Francesco Di Virgilio, Roberta Rizzo, Elena Adinolfi

**Affiliations:** ^1^ Department of Morphology, Surgery and Experimental Medicine, University of Ferrara, Ferrara, Italy; ^2^ Department of Medical Sciences, University of Ferrara, Ferrara, Italy; ^3^ Obstetrics and Gynaecology, School of Medicine, University of Geneve, Geneve, Switzerland

**Keywords:** P2X7, P2X7 489C>T polymorphism, HHV-6A infection, female infertility, HLA-G

## Abstract

The P2X7 receptor (P2X7R) is an ATP-gated ion channel known for its proinflammatory activity. Despite its participation in host defense against pathogens, the role played in viral infections, notably those caused by herpes viruses, has been seldom studied. Here we investigated the effect of P2X7R expression on human herpes virus 6 A (HHV-6A) infection of P2X7R-expressing HEK293 cells. We show that functional P2X7R increases while its blockade decreases viral load. Interestingly, HHV-6A infection was enhanced in HEK293 cells transfected with P2X7R cDNA bearing the gain of function 489C>T SNP (rs208294, replacing a histidine for tyrosine at position 155). The P2X7R 489C>T polymorphism correlated with HHV-6A infection also in a cohort of 50 women affected with idiopathic infertility, a condition previously shown to correlate with HHV-6A infection. None of the infertile women infected by HHV-6A was homozygote for 489CC genotype, while on the contrary HHV-6A infection significantly associated with the presence of the rs208294 allele. Levels of soluble human leukocyte antigen G (sHLA-G), a factor promoting embryo implant, measured in uterine flushings negatively correlated with the 489TT genotype and HHV-6A infection, while proinflammatory cytokines interleukins 1α (IL-1α), 1β (IL-1β), and 8 (IL-8) positively correlated with both the 489T allele presence and viral infection. Taken together these data point to the P2X7R as a new therapeutic target to prevent HHV-6A infection and the associated infertility.

## Introduction

The P2X7 receptor (P2X7R) ATP receptor is an ion channel belonging to the family of P2X receptors. The functional P2X7R is formed by a homomeric trimer whose subunit consists of a large extracellular loop including the agonist and antagonist binding sites, two alpha-helical transmembrane regions, and intracellular N and C-terminal peptides. Due to its widespread expression on immune cells and its ability to release cytokines the best-recognized function of P2X7R is to participate in inflammatory reactions. In the inflammatory microenvironment, ATP is released from dying cells as a damage-associated molecular pattern (DAMP) to activate the P2X7R and the following NLRP3-dependent release of several proinflammatory cytokines including interleukin (IL)-1β, IL-1α, and IL-18 (Di Virgilio et al., 2017; Orioli et al., 2017; Adinolfi et al., 2018). Accordingly, P2X7R can down-modulate the activity of anti-inflammatory molecules such as human leukocyte antigen (HLA)-G (Rizzo et al., 2009), an HLA class Ib molecule implicated in embryo protection and implantation *via* immune cells inhibition (Rizzo et al., 2007b). Moreover, P2X7R promotes hypoxia-inducible factor 1-α (HIF-1α) activation, vascular endothelial growth factor (VEGF) secretion, and neovascularization (Hill et al., 2010; Adinolfi et al., 2012; Amoroso et al., 2012; Amoroso et al., 2015). The human P2X7R is a highly polymorphic gene harboring over 13.000 single nucleotide polymorphisms (SNPs) the majority of which are nonsynonymous, intronic, or missense (Benzaquen et al., 2019). However, a small number of these SNPs has been shown to change receptor function, either as loss- (10) or gain- (3) of-function variants (Stokes et al., 2010; Bradley et al., 2011; De Marchi et al., 2016; Sluyter, 2017). Such diversity has been attributed to environmental pressure by infectious agents, such as mycobacterium tuberculosis, and association with chronic inflammatory diseases (Adinolfi et al., 2018). The most frequent human P2X7R SNP is 489C>T (rs208294, 47% frequency), leading the substitution of histidine 155, localized in the P2X7R ectodomain, with a tyrosine (H155Y) (Cabrini et al., 2005). Intriguingly, the CC genotype seems to be present mainly in the human receptor while the TT genotype, is the prevailing variant of the receptor in both mouse and rat (North and Surprenant, 2000). The 489C>T gain of function polymorphism has been recently associated with Alzheimer’s disease pathogenesis (Sanz et al., 2014) and in the accelerated release of proinflammatory cytokines observed in Lupus complicated with pericarditis (Hu et al., 2019). However, its implication in human disorders etiology remains understudied.

The P2X7R plays a major role in the response to infectious diseases, in particular, those caused by intracellular pathogens, *via* either direct effect on pathogen cell entry and survival or modulation of innate and adaptive immune responses (Adinolfi et al., 2018; Savio et al., 2018; Savio and Coutinho-Silva, 2019). In this context, the effect of P2X7R activation is not always beneficial or detrimental but depends upon the specific pathogen, its virulence and the severity of the infection (Savio et al., 2018). Therefore, the P2X7R can act as both host-protecting and infection-promoting factor. In viral infections, P2X7R inhibition protects against hepatitis, influenza, adenoviruses and HIV but it is detrimental in the case of vesicular stomatitis and Dengue viruses (Adinolfi et al., 2018; Savio et al., 2018). As per herpes viruses infections, limited evidence is available, showing P2X7R upregulation following cytomegalovirus infection (Zandberg et al., 2007). Human herpes virus 6 (HHV-6), a member of the Betaherpesvirinae subfamily and the causative agent of roseola infantum, has a wide cell tropism, albeit T cells are a preferred target. The acronym HHV-6 includes two distinct viruses, HHV-6A and -6B. HHV-6A recently emerged as a possible determinant of female idiopathic infertility, because infection by this virus alters endometrial immune cell responses and cytokine composition of the uterine milieu (Marci et al., 2016; Caselli et al., 2017; Bortolotti et al., 2019). Furthermore, HHV-6A infection causes a reduction in protein levels of the widely used phenotypic decidualization markers HLA-G (Rizzo et al., 2011) and mucin1 (MUC1) (McAuley et al., 2017). In this study, we explored the role played by the P2X7R in favoring HHV-6A infection and the associated infertility.

## Materials and Methods

### Cell Lines and Cultures

The acronyms HEK CC, HEK TT, and HEK mock are referring to HEK293 cells stably expressing human P2X7R-489CC, human P2X7R-489TT, and the empty expression vector PcDNA3, which were obtained as described in (Adinolfi et al., 2005; Cabrini et al., 2005). HEK293 cells were cultured in DMEM high glucose (Sigma), complemented with 10% heat-inactivated fetal bovine serum (FBS) (Euroclone, Milan, Italy), 100 U/ml penicillin (Euroclone), 100 mg/ml streptomycin (Euroclone), 1% non-essential amino acids (Sigma), and G418 sulfate 0,4 mg/ml (Sigma). HEC-1A endometrial epithelial cancer cell line (ATCC HTB-112) was cultured in McCoy’s 5a Medium (Sigma).

### P2X7R Immunofluorescence

Receptor’s surface expression was confirmed by immunocytochemistry (see **Supplementary Figure 1**). HEK mock, HEK CC and HEK TT cells were seeded on coverslips fixed with 4% paraformaldehyde for 15 min at 37°C, rinsed three times with phosphate-buffered saline (PBS), incubated in blocking buffer [PBS with 1% bovine serum albumin (BSA)] for 45 min at room temperature. Coverslips were then incubated with the antihuman P2X7R monoclonal antibody (mAb), previously characterized by Buell et al. (1998) kindly provided by Professor James Wiley (Florey Neuroscience Institutes, University of Melbourne, Australia) overnight at 4°C at a dilution of 1:50. Samples were rinsed and incubated with a TRICT conjugated anti-mouse antibody (T5393, Sigma-Aldrich) at a dilution of 1:500 for 1 h at room temperature. Fluorescence was visualized with a Leica DMI 4000B microscope equipped with 100× oil objective and images were acquired thanks to a Leica DFC 550 camera.

### HHV-6A Cell Infection and Assays for HHV-6 Detection

HEK293 and HEC-1A cells were infected with HHV-6A (strain U1102) cell-free virus inocula (Caselli et al., 2017) at a multiplicity of infection of 100 genome equivalents per 1 cell for 2 h at 37°C. HHV-6A antigen expression was analyzed by immunofluorescence 5 days post-infection (dpi) with a mouse mAb that recognized the glycoprotein gp116 (late antigen) of HHV-6A (ABI, Columbia, MD, United States), as previously described (Caselli et al., 2017).

HHV-6A DNA extraction and analysis was performed as previously described (Rizzo et al., 2019). Briefly, real-time quantitative polymerase chain reaction (qPCR) specific for the U94 gene was used to determine HHV-6A DNA presence and load. Positive samples were considered those in which 1 µg of cellular DNA harbored more than 100 copies of viral DNA. The following set of primers/probe was used for qPCR: HHV6 U94(+) (5′-GAG CGC CCG ATA TTA AAT GGA T-3′); HHV6 U94(−) (5′-GCT TGA GCG TAC CAC TTT GCA-3′); HHV6 U94 PROBE (5′-FAM-CTG GAA TAA TAA AAC TGC CGT CCC CAC C-TAMRA-3′). The standard curve was generated by amplification of a plasmid containing the targeted HHV-6 sequences. Human RNase P or beta-actin housekeeping genes were used as a control.

RNA cell extraction was performed with the RNeasy kit (Qiagen, Hilden, Germany). The absence of contaminant DNA in the extracted RNA was assured by DNase treatment and control β-actin PCR without retrotranscription reverse transcription (Caselli et al., 2017; Rizzo et al., 2017). The analysis of virus transcripts was performed by RNA reverse transcription with the RT2 First-strand kit (Qiagen, Hilden, Germany) using cDNA aliquots obtained from 200 ng RNA (Menegazzi et al., 1999; Caselli et al., 2012). The specific primers used to amplify HHV-6A U42 were: forward 3′ACGATGGACATGGCTTGTTG5′; reverse 3′ACCTTACAACGGAGACGCC5′ (Caselli et al., 2012). The methods had a 6-log dynamic range and a sensitivity of 20 copies/ml. Each sample was run in duplicate.

### HLA-G Detection

Ten microliters of cell culture supernatants or uterine flushing samples were assayed for soluble HLA-G using a bead array Bio-Plex system (BioRad, CA, USA) with anti-HLA-G MoAb (G233; Exbio, Czech Republic) conjugated beads, as previously reported (Rizzo et al., 2007a).

### CD46 Flow Cytometry

CD46 surface expression data were analyzed using FACS CantoII flow cytometer (BD, Milan, Italy) and FlowJo LLC analysis software (Ashland, Oregon, USA). Cells (1 ×10^5^) were labeled with CD46-PE (R&D Systems, Italy) or matched isotype controls. Ten thousand events per cell type were acquired.

### Clinical Samples

Endometrial specimens were obtained by biopsy from patients admitted for tubal patency assessment by Hystero-sono contrast sonography at secretory stage of the menstrual cycle. The endometrium was prepared as previously described (Marci et al., 2016). Endometrial epithelial cells were collected from the Ficoll-Paque-medium interface using BerEP4-coated magnetic Dynabeads system (Dynal Biotech, Oslo, Norway). The sorted epithelial cells were typed for HHV-6A or B identification. DNA extraction and analysis were performed as previously described (Caselli et al., 2012). Above-mentioned qPCR was used to determine HHV-6 DNA presence and load. All samples were randomly and blindly investigated and we obtained enough material the analysis was repeated twice in a randomized and blinded fashion at a distant time set from the first determination. HHV-6A or B identification was performed as reported previously (Caselli et al., 2012), by restriction enzyme digestion of the U31 nested PCR amplification product and visualization of the digestion products on ethidium bromide-stained agarose gel after electrophoresis migration.

Uterine flushing samples were obtained from the same patients including only 21–38 years old women, with regular menstrual cycle (24–35 days), a body mass index (BMI) ranging between 18 and 26 kg/m^2^, follicle-stimulating hormone (FSH; days 2–3 of the menstrual cycle) < 10 mUI/ml, 17-β-estradiol < 50 pg/ml (days 2–3 of the menstrual cycle), normal karyotype. Women that presented endometritis, endometriosis, tubal factor, ovulatory dysfunction, anatomical uterine pathologies, and recurrent miscarriage were excluded. Uterine flushing was performed with a 14-gauge Foley three-way balloon catheter (Eschmann) inflating an appropriate (5 ml) amount of sterile physiologic saline solution (Rizzo et al., 2017). Genomic DNA for P2X7R polymorphism assessment and circulating HHV6-A and B detection was isolated from whole blood using the QIAamp DNA Blood Mini kit (QIAGEN, Hilden, Germany). This study was approved by the “University-Hospital of Ferrara Ethics Committee.” All subjects gave written informed consent in accordance with the Declaration of Helsinki.

### P2X7R 489C>T Polymorphism Analysis

The presence of 489C>T SNP was determined by real-time PCR using an allelic discrimination TaqMan MGB probe technique with a Step One Real-Time PCR thermal cycler (Applied Biosystem) as previously described (Cabrini et al., 2005). Briefly, genomic DNA (100 ng) was added to the PCR master mix (TaqMan Universal PCR Master Mix, Applied Biosystems) in the presence of validated primers and probes (Applied Biosystems, ID number: C:_3019032_1_). Following 40 PCR cycles (15 s at 95°C for denaturation and 1 min at 60°C annealing plus elongation), genotype was assigned to each sample analyzing the fluorescent signal.

### Cytokines and Growth Factor Evaluation in Uterine Flushing Samples

Cytokines were analyzed in uterine flushing samples using Cyraplex assay (Aushon, distributed by Tema Ricerca, Bologna, Italy) according to the manufacturers’ instructions. VEGF levels were assessed by PicoKine™ ELISA kit (Boster, distributed by Tema Ricerca, Bologna, Italy).

### Measurement of Cytosolic Ca^2+^

Changes in the intracellular HEC-1A Ca^2+^ concentration were measured with the fluorescent indicator Fura-2/acetoxymethylester (Fura-2/AM), using a Cary Eclipse Fluorescence Spectrophotometer (Agilent Technologies, Milan, Italy). Cells were seeded on a coverslip and maintained at 37°C in a 5% CO_2_ humidified incubator until confluence. The confluent monolayers were loaded with Fura-2/AM (4 μM) in standard saline solution: 125 mM NaCl, 5 mM KCl, 1 mM MgSO_4_, 1 mM NaH_2_PO_4_, 20 mM HEPES, 5.5 mM glucose, 5 mM NaHCO_3_, 1 mM CaCl_2_, and 250 μM sulfinpyrazone (Sigma-Aldrich). Incubation was performed at 37°C for 20 min. Cells were then washed and changes of intracellular [Ca^2+^] were determined after stimulation with 300 μM 2′-(3′)-0-(4-benzoylbenzoyl) ATP (Sigma-Aldrich) following, whenever required, by 10-min incubation with AZ10606120, in a thermostated cuvette under stirring, with a 340/380 excitation ratio at an emission wavelength of 505 nm.

### Statistics and Data Availability

Data were analyzed for normality by Kolmogorov–Smirnov test. All the variables resulted normally distributed. Therefore, analysis of variance (ANOVA) and two-tailed Student’s t tests were used to compare most of the variables. *X* ^2^ test and Fisher exact test were used to compare allelic and genotypic frequencies and positivity for HHV6-A (**Table 1**) and sHLA-G (**Figure 2B**) respectively. P-values lower than 0.05 were considered statistically significant. All statistic evaluations were performed thanks to GraphPad Prism software (GraphPad, La Jolla, California, USA). The raw data supporting the conclusions of this manuscript will be made available by the authors, upon reasonable request, to any qualified researcher.

**Table 1:** Allele and genotype frequencies of P2X7R polymorphism 489C>T in infertile women population (n = 50) subdivided according to HHV-6A infection into HHV-6A negative women (HHV-6A−, n = 39) and HHV-6A positive women (HHV-6A+, n = 11).

Polymorphism 489C>T	Allele frequency %		Genotype frequency %		
	C	T	CC	CT	TT
Infertile women* (n = 50)	0.58	0.42	32	52	16
HHV-6A infection					
HHV-6A -^$^ (n = 39)	0.65	0.35	41	49	10
HHV-6A +*^$^ (n = 11)	0.32	0.68	0	63	37

*p = 0.0358 HHV-6A+ women vs overall population (*X^2^* test).

^$^p = 0.0021 HHV-6A - women vs HHV-6A+ women (*X^2^* test).

The genotype frequencies of HHV-6A− and HHV-6A+ women were compared between them and with those of the total tested population. The genotype frequencies were found significantly different in HHV-6A+ women as compared to the overall population (p = 0.0358) and HHV-6A− women (p = 0.0021). While, no significant difference between HHV-6A− women and the overall population was found (p = 0.3911). P-value was calculated with the *X* ^2^ test. HHV-6A negative with P2X7R 489 genotype CC (n = 16), HHV-6A negative with P2X7R 489 genotype CT (n = 19), HHV-6A negative with P2X7R 489 genotype TT (n = 4), HHV-6A positive with P2X7R 489 genotype CT (n = 7), HHV-6A positive with P2X7R 489 genotype TT (n = 4).

## Results

### P2X7R Favors HHV-6A Infection in HEK293 Cells

**Figure 1:**
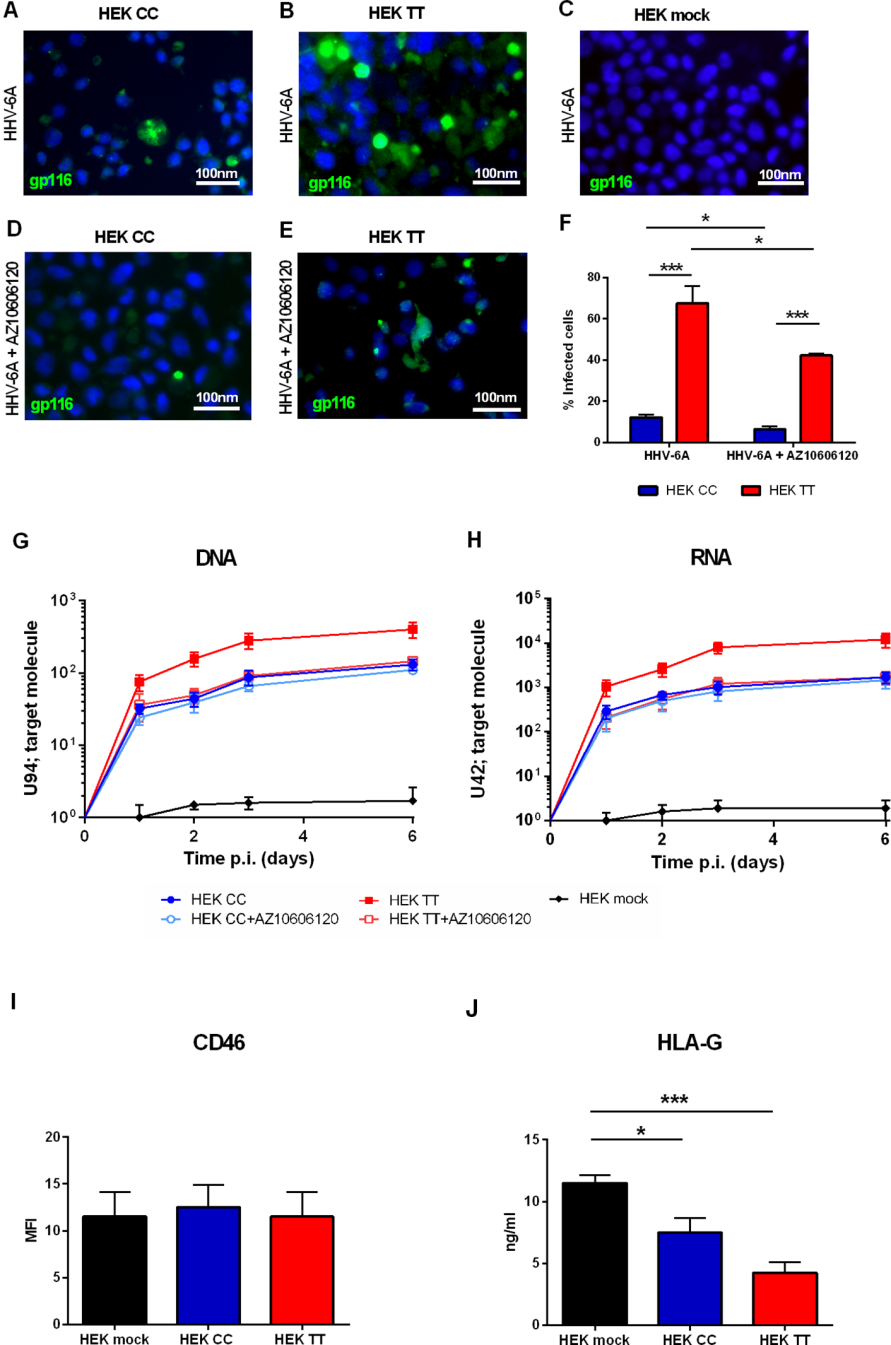
**(A–H)** HHV6-A infection of HEK293 cells. Cells were infected with 100 genome equivalents per cell of HHV-6A for 2 h at 37°C in the presence or not of P2X7R antagonist AZ10606120 (2 µM). **(A–E)** Fluorescence pictures of HHV-6A infected HEK CC **(A, D)**, HEK TT **(B, E)**, and HEK mock cells **(C)** stained with anti-gp116 mAb. **(F)** Percentage of HEK CC and HEK TT cells positive for HHV-6A gp116 protein in the presence or not of P2X7R antagonist AZ10606120. HHV-6A positivity significantly differs among groups [p < 0.0001, analysis of variance (ANOVA) test]. Data for each group are shown as the mean ± SEM of four experiments. Groups were compared with each other using two-tailed Student’s t-test, *p < 0.05, ***p < 0.001. HHV6-A U94 DNA **(G)** and U42 RNA levels **(H)**. U94 DNA and U42 RNA were quantified by polymerase chain reaction (PCR) and quantitative PCR (qPCR) as reported in *Materials and Methods*. **(I)** Mean fluorescence intensity (MFI) of HHV-6A membrane receptor CD46 on HEK mock, HEK CC, and HEK TT cells. Expression of CD46 is not significantly different among groups (p = 0.34, ANOVA test). **(J)** Human leukocyte antigen G (HLA-G) concentration was evaluated in HEK mock, HEK CC, and HEK TT cell culture supernatants and differed among the samples (p = 0.0012; ANOVA test). Data for each group are shown as the mean ± SEM of four experiments. Groups were further compared with each other using two-tailed Student’s t-test, *p < 0.05, ***p < 0.001.

HEK293 human embryonic kidney cells are an interesting model for P2X scholars as they do not express a functional P2X7R receptor or any other member of the P2X family and can be easily transfected to induce the overexpression of virtually all P2X receptors (Adinolfi et al., 2005). Therefore we used HEK293 stably transfected cell clones carrying either a) the WT P2X7R SNP, i.e. the 489CC genotype (HEK CC), or b) the gain of function P2X7R SNP, i.e. the 489TT genotype (HEK TT) or the empty vector (HEK mock) and challenged them with HHV-6A virions. **Figure 1** shows that P2X7R expression strongly increases HHV-6A infection in HEK293 cells. The viral infection is dependent upon the 489 P2X7R genotype as HEK TT are infected in a significantly higher percentage if compared to HEK CC (**Figures 1A, B, F**). Moreover, both late gene U94 DNA and immediate early U42 gene RNA levels are 1 log higher in HEK TT in comparison with HEK CC and the expression of both receptors variants leads to an increase of about 2 logs of HHV6-A genes as compared to HEK mock cells (**Figures 1G, H**). P2X7R antagonism with AZ10606120 strongly reduced HHV-6A infection in HEK TT cells as demonstrated by both immunofluorescence (**Figures 1B, E, F**) and gene evaluation data (**Figures 1G, H**). However, HEK CC HHV-6A positivity was only slightly reduced by the receptor antagonist (**Figures 1G, H**) and viral DNA and RNA expression, although presenting a clear trend toward reduction (~20%) were not significantly affected (**Figures 1G, H**). Since HHV-6A entry into target cells is determined by CD46 surface expression (Santoro et al., 1999), we evaluated CD46 levels on the surface of HEK293 transfected with the different P2X7R variants (**Figure 1I**). However, P2X7R expression or genotype did not affect the expression of the HHV-6A receptor CD46 (**Figure 1I**) (p = 0.34; ANOVA test), supporting a role for P2X7R in the control of viral infection. Since we know that both P2X7R and HHV-6 can affect HLA-G expression (Rizzo et al., 2009; Caselli et al., 2015), we measured HLA-G concentration in HEK293 culture supernatants (**Figure 1J**). Secretion of HLA-G was different between the different samples (p = 0.0012; ANOVA test). Soluble HLA-G (sHLA-G) inversely correlated with P2X7R expression and was dependent upon 489 SNP with the 489TT genotype associated with a larger reduction of HLA-G release (p < 0.001; Student t-test) (**Figure 1J**).

### P2X7R 489CC Protects Against HHV-6A Infection and Affects the Uterine Cytokine Milieu in Infertile Women

**Figure 2:**
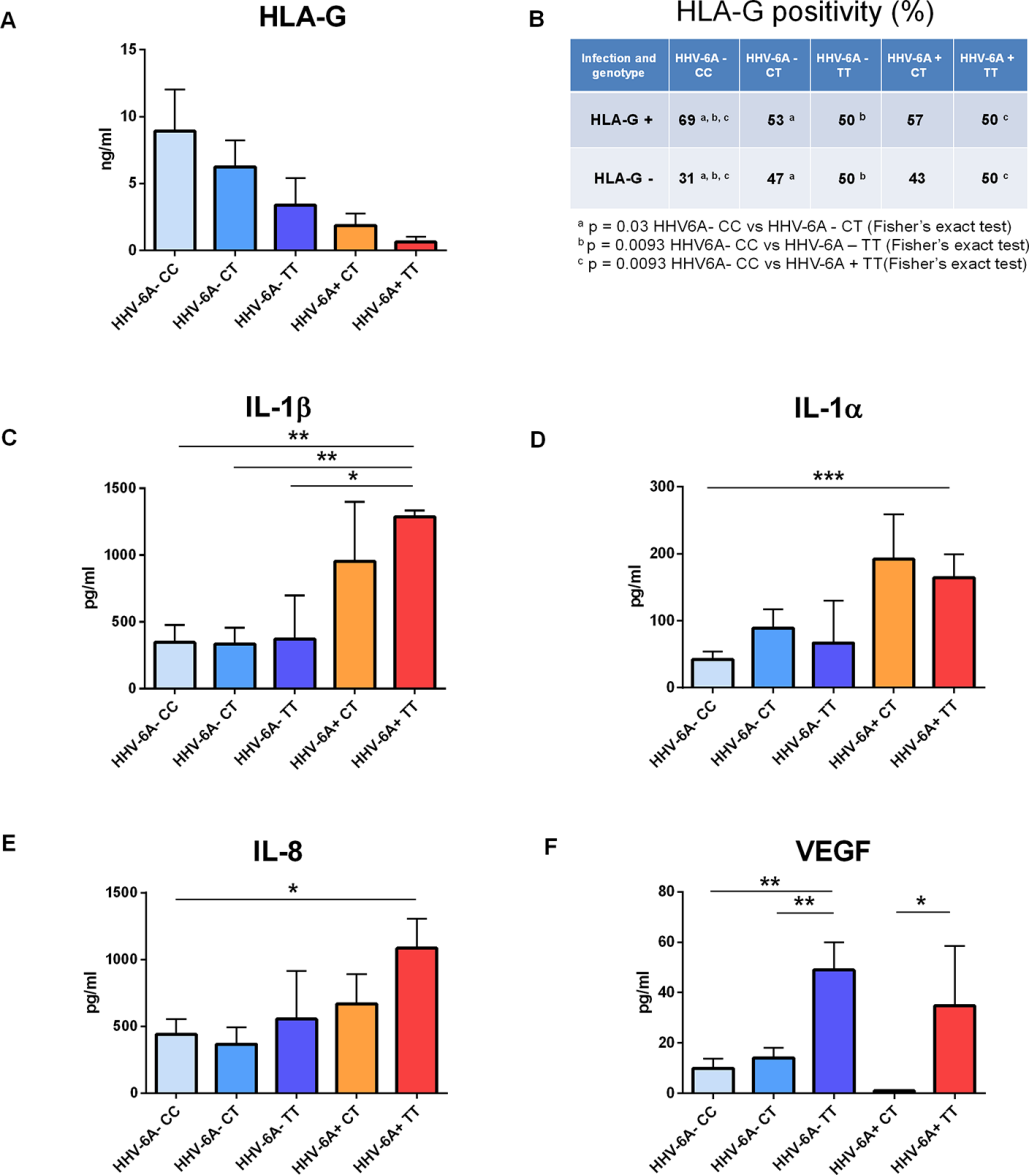
Correlation between P2X7R polymorphism 489C>T and uterine milieu composition of infertile women infected or not with HHV-6A virus. **(A–F)** HLA-G, interleukin (IL)-1β, IL-1α, IL-8, and vascular endothelial growth factor (VEGF) concentrations were evaluated in uterine flushing samples from infertile women (n = 50) and analyzed according to P2X7R 489 genotype and HHV-6A infection as follows: HHV-6A negative with P2X7R 489 genotype CC (n = 16), HHV-6A negative with P2X7R 489 genotype CT (n = 19), HHV-6A negative with P2X7R 489 genotype TT (n = 4), HHV-6A positive with P2X7R 489 genotype CT (n = 7), and HHV-6A positive with P2X7R 489 genotype TT (n = 4). **(A)** HLA-G concentrations are not significantly different among the groups analyzed (p = 0.3196, ANOVA test). However, when analyzing the percentage of samples positive for HLA-G **(B)** according to P2X7R 489C>T polymorphism and HHV-6A infection there is a significant decrease of positivity of HLA-G in women positive for the virus and carrying 489T allele **(B)** (p < 0.05, Fisher’s exact test). **(C–F)** Proinflammatory cytokines and VEGF concentrations are affected by P2X7R genotype and HHV-6A positivity. IL-1β **(C)**, IL-1α **(D)**, IL-8 **(E)**, and VEGF **(F)** concentrations are different among the samples by ANOVA test (p = 0.0132, p = 0.03, p = 0.045, p = 0.012, respectively). Data for each group are shown as the mean ± SEM and compared with each other using two-tailed Student’s t-test, *p < 0.05, **p < 0.01, and ***p < 0.001.

**Figure 3:**
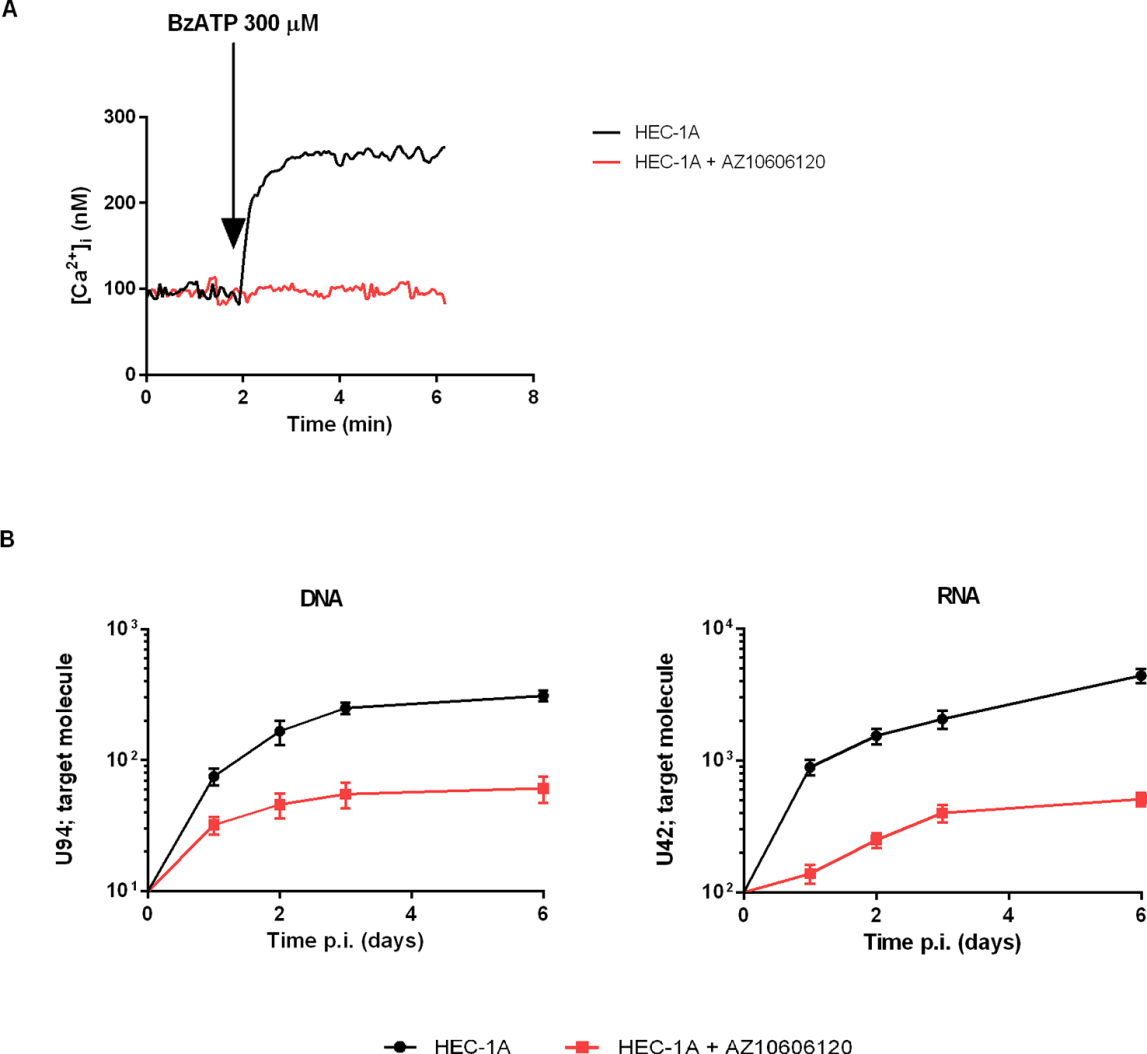
Demonstration of functional P2X7R in the endometrial cancer cell line HEC-1A and infection with HHV-6A virus. HEC-1A carries the P2X7R 489TT genotype. **(A)** Representative trace showing P2X7R-dependent intracellular calcium increase following cells stimulation with BzATP (300 µM) alone (black) or in the presence of P2X7R antagonist AZ10606120 (2 μM, red). **(B)** HHV-6A infection in HEC-1A cells. Cells were infected with 100 genome equivalents per 1 cell of HHV-6A for 2 h at 37°C. U94 DNA and U42 RNA were quantified by real-time PCR and RT-PCR as reported in *Materials and Methods*. Black control cells, red cells pretreated with 2 μM AZ10606120.

We next investigated the association of P2X7R 489C>T SNP with HHV-6A infection in a pathological condition. HHV-6A infection and low HLA-G levels in the uterine environment were recently associated with female idiopathic infertility (Marci et al., 2016; Bortolotti et al., 2019), therefore we analyzed the frequency of 489C>T SNP and the presence of HHV-6A DNA in endometrial biopsies from a cohort of 50 women with idiopathic infertility. The endometrial biopsies were analyzed for the presence of HHV-6A and B infection. More than 20% (11/50) women with idiopathic infertility were positive for HHV-6A DNA in their endometrial epithelial cells. HHV-6B DNA was not present in all the endometrial biopsies as previously reported (Marci et al., 2016; Bortolotti et al., 2019). The average viral load in endometrial epithelial cells from HHV-6A positive infertile women was 490.000 copies/µg of cellular DNA (range 698.000–236.000 copies/µg DNA), corresponding to about 4 copies of viral DNA per diploid cell. As a control, we evaluated HHV-6A and HHV-6B DNA presence in the peripheral blood mononuclear cells (PBMCs) of our population. While we confirmed the previously reported 25% positivity for HHV-6B virus (Caselli, 2007) HHV6-A was not present in PBMCs from our population (not shown). **Table 1** shows a negative association between HHV-6A infection and 489 C homozygosis with the number of infected women significantly growing with the presence of the minor gain of function allele 489T. These data strongly suggest an association between the rs208294 allele and the susceptibility to HHV6-A infection in idiopathic infertile women. Analysis of HLA-G concentration in uterine flushings from the same women revealed a clear tendency toward a decrease of HLA-G from HHV-6A negative/489CC to HHV-6A positive/489TT subjects (**Figure 2A**). The high variability in HLA-G concentration found in our population was due to the presence of flushing samples where HLA-G was either absent or below the threshold for detection. Therefore, we re-analyzed the population comparing the percentage of HLA-G positive versus HLA-G negative flushing samples by fisher exact test (**Figure 2B**). This analysis highlighted statistically significant differences between women clustered according to the 489 genotype and HHV-6A status of infection, with the HHV-6A negative/489CC subjects showing the highest HLA-G percentages as compared to infected and 489TT women (**Figure 2B**). On the contrary, proinflammatory cytokines and chemokines showed a tendency to increase in the presence of 489T allele and HHV-6A infection (**Figures 2C–E**). As expected, based on the central role played by P2X7R in its maturation and secretion, IL-1β flushing concentrations differed between the cohorts (p = 0.0132; ANOVA test), tending to increase in women with the 489T allele, reaching statistically significantly high concentrations in subjects both homozygous for 489T and infected with HHV-6A (p < 0.001; Student t-test) (**Figure 2C**). A similar trend was also observed for IL-1α (p = 0.03; ANOVA test) (**Figure 2D**) and IL-8 (p = 0.045; ANOVA test) (**Figure 2E**). Finally VEGF concentration in the flushing samples solely associated with 489TT genotype independently of the infection status (p = 0.012; ANOVA test) (**Figure 2F**). Other cytokines tested, including IFNγ, IL-4, IL-6, IL-10, IL-12p70, and TNF-α, were either undetectable or present at low concentrations and did not significantly differ within the tested populations (**Supplementary Figure 2**). As a proof of concept of the expression of P2X7R by endometrial epithelial cells, we analyzed HEC-1A endometrial epithelial cell line. Interestingly, a functional P2X7R, homozygous for the 489T allele, was also found in these cells (**Figure 3A**). We observed significant infection of these cells that was reduced by P2X7R antagonism with AZ10606120 (**Figure 3B**).

## Discussion

Purinergic signaling is known to participate in viral infection and replication, and in anti-viral inflammatory responses and, consequently, purinergic receptors have been proposed as targets for antiviral therapy (Ferrari et al., 2018; Savio et al., 2018; Zhang et al., 2019). Among P2 purinergic receptors, the P2X7R is one of the best candidates for such intervention as its activity in promoting or inhibiting viral infection, or alternatively its ability to support antiviral responses was widely demonstrated. Furthermore, several potent and selective inhibitors have been developed over time (De Marchi et al., 2016; Di Virgilio et al., 2017; Adinolfi et al., 2018; Savio et al., 2018). In the present study, we showed a predisposing role for the P2X7R in mediating HHV-6A infection. This activity was not due to changes in the expression of the known viral receptor CD46 since its levels remained unaltered in P2X7R expressing cells as compared to mock control. In support of a main role of a functional P2X7R, we also showed that a selective P2X7R blocker, AZ10606120 (De Marchi et al., 2019), reduced HHV-6A infection especially in HEK TT P2X7R-expressing cells. The identification of new drugs that can counteract HHV-6 infection is of extreme importance. Increased awareness of diseases associated with HHV-6 acute infection, in both immunocompetent and immunocompromised patients, has spurred interest in the development of effective treatments for HHV-6-mediated diseases. A number of drugs used for cytomegalovirus infection have shown *in vitro* efficacy against HHV-6. Dozens of case reports and several small clinical studies have demonstrated the efficacy of different compounds against HHV-6 infection. However, no drug has yet been approved for use in humans. The discovery that P2X7R expression facilitates HHV-6A infection opens an entirely novel avenue for anti-viral drug therapy. Our data show that HHV-6A infection *in vitro* and *in vivo* depends on P2X7R function as it is enhanced in the presence of the gain of function 489TT polymorphic variant. Previous studies showed that this polymorphism increases P2X7R activity as both a small ion channel and large cation permeation pathway (Cabrini et al., 2005; Roger et al., 2010) and that this phenotype is possibly linked to increased cell surface expression of P2X7R (Bradley et al., 2011). These data suggest that the SNP could favor viral entry either due to P2X7R membrane pore formation or to viral direct interaction on the cell surface. Another possible explanation for P2X7R-mediated HHV6-A cellular entry could be receptor-dependent phagocytic uptake (Gu and Wiley, 2018; Ou et al., 2018). Moreover, P2X7R-dependent increased HHV-6A infection inversely correlated with the secretion of HLA-G from HEK293 cells thus suggesting also an effect on the activation of the immune system (Eliassen et al., 2017). Data from HEK293 cells were confirmed by the complete absence of the 489CC homozygous genotype in primary infertile women bearing an endometrial HHV-6A infection (**Table 1**). To further support the role of the T allele in promoting HHV-6A infection, infection rates increase in parallel with 489T allele (**Table 1**). In agreement with the findings in HEK293 cells, also in the patient cohort the 489TT genotype correlated with decreased presence of sHLA-G in uterine flushing samples (**Figures 2A, B**). This observation is in keeping with the known modulatory effect of HHV-6A infection on HLA-G expression (Rizzo et al., 2018). HHV-6-induced increase of HLA-G is likely mediated by human transcription factor ATF3, that binds a consensus sequence on the HLA-G promoter (Rizzo et al., 2018). The largest difference in cytokines, chemokines and growth factors in uterine flushings was measured between the 489 CC/HHV-6A negative and the 489TT/HHV-6A positive subjects. 489TT/HHV-6A positive women also showed higher levels of IL-1β, IL-1α, and IL-8 (CXCL8) suggesting that the 489TT gain-of-function variant not only favors virus entry but also triggers a wide inflammatory response that might contribute to female infertility (Tsimis et al., 2017). It is therefore tempting to speculate that P2X7R-mediated HHV-6A infection and the associated female-infertility might have been a drive for negative selection of the 489T variant in favor of the 489C variant, and a possible mechanism that has favored this hypomorphic allele in humans as opposed to rodents, which are not infected by HHV-6 (Reynaud and Horvat, 2013) and where the 489T is the common allele (Surprenant et al., 1996; Rassendren et al., 1997; Chessell et al., 1998). Our data also confirm that the 489T allele increases P2X7R channel and pore function (Cabrini et al., 2005; Roger et al., 2010) as well as IL-1, IL-8, and VEGF secretion (Orioli et al., 2017). P2X7R-mediated modulation of the uterine microenvironment could be due to both immune and endometrial cells, as these latter do express a functional P2X7R. Interestingly, P2X7R antagonism strongly reduced HHV6-A infection rate in HEC-1A endometrial cells (**Figure 3**) opening the way to the application of P2X7R targeting drugs also to this cellular subset.

Our data suggest a possible therapeutic application of P2X7R antagonists in young idiopathic infertile women. P2X7R blockers could be ideally locally administered in the endometrium to prevent or lower HHV-6A infection. Additionally, P2X7R blockade during the implantation window should help to reconstitute endometrial receptivity on one hand *via* induction of HLA-G and on the other *via* reduction of proinflammatory cytokines, chemokines, and VEGF. The recent use of P2X7R antagonists in several clinical trials guarantees their safety and a quick transfer to the clinical practice (De Marchi et al., 2016; Di Virgilio et al., 2017; Park and Kim, 2017; Di Virgilio et al., 2018), making them in principle useful therapeutic tools also in other HHV-6A-associated diseases such as roseola infantum, autoimmune diseases, chronic fatigue syndrome, encephalitis, and Alzheimer’s disease (Rizzo et al., 2019).

## Data Availability Statement

The raw data supporting the conclusions of the manuscript will be made available by the authors, upon reasonable request, to any qualified researcher.

## Ethics Statement

The studies involving human participants were reviewed and approved by the University Hospital of Ferrara Ethics Committee. The patients/participants provided their written informed consent to participate in this study.

## Author Contributions

AP and DB performed most of the experimental work, helped with paper writing and experimental design. RM was responsible for patients selection and clinical evaluation. SF and EDM helped with PCR and *in vitro* experiments. EC helped with viral infections and experimental design. FDV participated in experimental design and manuscript writing. EA and RR were responsible for overall study conception, experimental design, data interpretation, manuscript writing, and final approval. AP and DB equally contributed to the study. EA and RR equally contributed to this study.

## Funding

This study was funded by an Italian Association for Cancer Research Investigator Grant to EA (AIRC, IG 16812 and IG22837) and institutional funds from the University of Ferrara. The funders had no role in the design of the study, in the execution, analyses, interpretation of the data, and decision to submit results.

## Conflict of Interest

The authors declare that the research was conducted in the absence of any commercial or financial relationships that could be construed as a potential conflict of interest.

## Supplementary Material

Supplementary Figure 1 P2X7R surface expression determined by immunofluorescence with anti-P2X7R mAb in **(A)** HEK mock, **(B)** HEK CC and **(C)** HEK TT.

Supplementary Figure 2
**(A)** IFN-γ, **(B)** IL-4, **(C)** IL-6, **(D)** IL-10, **(E)** IL-12p70 and **(F)** TNF-α concentrations were evaluated in uterine flushing samples from infertile women (n = 50) and analyzed according to P2X7R 489 genotype and HHV-6A infection as follows: HHV-6A negative with P2X7R 489 genotype CC (n = 16), HHV-6A negative with P2X7R 489 genotype CT (n = 19), HHV-6A negative with P2X7R 489 genotype TT (n = 4), HHV-6A positive with P2X7R 489 genotype CT (n = 7), HHV-6A positive with P2X7R 489 genotype TT (n = 4). They did not significantly differ within the tested populations by ANOVA test (p = 0.2503, p = 0.7628, p = 0.2436, p = 0.6017, p = 0.7609, p = 0.5105, respectively).

## References

[B1] Adinolfi E. Callegari M. G. Ferrari D. Bolognesi C. Minelli M. Wieckowski M. R. (2005). Basal activation of the P2X7 ATP receptor elevates mitochondrial calcium and potential, increases cellular ATP levels, and promotes serum-independent growth. Mol. Biol. Cell 16 (7), 3260–3272. 10.1091/mbc.e04-11-1025 15901833 PMC1165409

[B2] Adinolfi E. Raffaghello L. Giuliani A. L. Cavazzini L. Capece M. Chiozzi P. (2012). Expression of P2X7 receptor increases *in vivo* tumor growth. Cancer Res. 72 (12), 2957–2969. 10.1158/0008-5472.CAN-11-1947 22505653

[B3] Adinolfi E. Giuliani A. L. De Marchi E. Pegoraro A. Orioli E. Di Virgilio F. (2018). The P2X7 receptor: a main player in inflammation. Biochem. Pharmacol. 151, 234–244. 10.1016/j.bcp.2017.12.021 29288626

[B4] Amoroso F. Falzoni S. Adinolfi E. Ferrari D. Di Virgilio F. (2012). The P2X7 receptor is a key modulator of aerobic glycolysis. Cell Death Dis. 3, e370. 10.1038/cddis.2012.105 22898868 PMC3434661

[B5] Amoroso F. Capece M. Rotondo A. Cangelosi D. Ferracin M. Franceschini A. (2015). The P2X7 receptor is a key modulator of the PI3K/GSK3beta/VEGF signaling network: evidence in experimental neuroblastoma. Oncogene 34 (41), 5240–5251. 10.1038/onc.2014.444 25619831

[B6] Benzaquen J. Heeke S. Janho Dit Hreich S. Douguet L. Marquette C. H. Hofman P. (2019). Alternative splicing of P2RX7 pre-messenger RNA in health and diseases: myth or reality? BioMed. J. 42 (3), 141–154. 10.1016/j.bj.2019.05.007 31466708 PMC6717933

[B7] Bortolotti D. Gentili V. Rotola A. Cultrera R. Marci R. Di Luca D. (2019). HHV-6A infection of endometrial epithelial cells affects immune profile and trophoblast invasion. Am. J. Reprod. Immunol. 82 (4), e13174. 10.1111/aji.13174 31338899

[B8] Bradley H. J. Baldwin J. M. Goli G. R. Johnson B. Zou J. Sivaprasadarao A. (2011). Residues 155 and 348 contribute to the determination of P2X7 receptor function *via* distinct mechanisms revealed by single-nucleotide polymorphisms. J. Biol. Chem. 286 (10), 8176–8187. 10.1074/jbc.M110.211284 21205829 PMC3048704

[B9] Buell G. Chessell I. P. Michel A. D. Collo G. Salazzo M. Herren S. (1998). Blockade of human P2X7 receptor function with a monoclonal antibody. Blood 92 (10), 3521–3528. 10.1182/blood.V92.10.3521 9808543

[B10] Cabrini G. Falzoni S. Forchap S. L. Pellegatti P. Balboni A. Agostini P. (2005). A His-155 to Tyr polymorphism confers gain-of-function to the human P2X7 receptor of human leukemic lymphocytes. J. Immunol. 175 (1), 82–89. 10.4049/jimmunol.175.1.82 15972634

[B11] Caselli E. Di Luca D. (2007) Molecular biology and clinical associations of roseoloviruses human herpesvirus 6 and human herpesvirus 7. New Microbiol. 30, 173–187. 17802896

[B12] Caselli E. Zatelli M. C. Rizzo R. Benedetti S. Martorelli D. Trasforini G. (2012). Virologic and immunologic evidence supporting an association between HHV-6 and Hashimoto’s thyroiditis. PloS Pathog. 8 (10), e1002951. 10.1371/journal.ppat.1002951 23055929 PMC3464215

[B13] Caselli E. Campioni D. Cavazzini F. Gentili V. Bortolotti D. Cuneo A. (2015). Acute human herpesvirus-6A infection of human mesothelial cells modulates HLA molecules. Arch. Virol. 160 (9), 2141–2149. 10.1007/s00705-015-2490-3 26085284

[B14] Caselli E. Bortolotti D. Marci R. Rotola A. Gentili V. Soffritti I. (2017). HHV-6A infection of endometrial epithelial cells induces increased endometrial NK Cell-mediated cytotoxicity. Front. Microbiol. 8, 2525. 10.3389/fmicb.2017.02525 29326672 PMC5736868

[B15] Chessell I. P. Simon J. Hibell A. D. Michel A. D. Barnard E. A. Humphrey P. P. (1998). Cloning and functional characterisation of the mouse P2X7 receptor. FEBS Lett. 439 (1-2), 26–30. 10.1016/s0014-5793(98)01332-5 9849870

[B16] De Marchi E. Orioli E. Dal Ben D. Adinolfi E. (2016). P2X7 Receptor as a Therapeutic Target. Adv. Protein Chem. Struct. Biol. 104, 39–79. 10.1016/bs.apcsb.2015.11.004 27038372

[B17] De Marchi E. Orioli E. Pegoraro A. Sangaletti S. Portararo P. Curti A. (2019). The P2X7 receptor modulates immune cells infiltration, ectonucleotidases expression and extracellular ATP levels in the tumor microenvironment. Oncogene 38 (19), 3636–3650. 10.1038/s41388-019-0684-y 30655604 PMC6756114

[B18] Di Virgilio F. Dal Ben D. Sarti A. C. Giuliani A. L. Falzoni S. (2017). The P2X7 receptor in infection and inflammation. Immunity 47 (1), 15–31. 10.1016/j.immuni.2017.06.020 28723547

[B19] Di Virgilio F. Sarti A. C. Falzoni S. De Marchi E. Adinolfi E. (2018). Extracellular ATP and P2 purinergic signalling in the tumour microenvironment. Nat. Rev. Cancer 18 (10), 601–618. 10.1038/s41568-018-0037-0 30006588

[B20] Eliassen E. Di Luca D. Rizzo R. Barao I. (2017). The Interplay between Natural killer cells and human herpesvirus-6. Viruses 9 (12). 10.3390/v9120367 PMC5744142 29194419

[B21] Ferrari D. Idzko M. Muller T. Manservigi R. Marconi P. (2018). Purinergic signaling: a new pharmacological target against viruses? Trends Pharmacol. Sci. 39 (11), 926–936. 10.1016/j.tips.2018.09.004 30292585

[B22] Gu B. J. Wiley J. S. (2018). P2X7 as a scavenger receptor for innate phagocytosis in the brain. Br. J. Pharmacol. 175 (22), 4195–4208. 10.1111/bph.14470 30098011 PMC6193880

[B23] Hill L. M. Gavala M. L. Lenertz L. Y. Bertics P. J. (2010). Extracellular ATP may contribute to tissue repair by rapidly stimulating purinergic receptor X7-dependent vascular endothelial growth factor release from primary human monocytes. J. Immunol. 185 (5), 3028–3034. 10.4049/jimmunol.1001298 20668222 PMC3156583

[B24] Hu S. Yu F. Ye C. Huang X. Lei X. Dai Y. (2019). The presence of P2RX7 single nuclear polymorphism is associated with a gain of function in P2X7 receptor and inflammasome activation in SLE complicated with pericarditis. Clin. Exp. Rheumatol. 31376246

[B25] Marci R. Gentili V. Bortolotti D. Lo Monte G. Caselli E. Bolzani S. (2016). Presence of HHV-6A in endometrial epithelial cells from women with primary unexplained infertility. PloS One 11 (7), e0158304. 10.1371/journal.pone.0158304 27367597 PMC4930213

[B26] McAuley J. L. Corcilius L. Tan H. X. Payne R. J. McGuckin M. A. Brown L. E. (2017). The cell surface mucin MUC1 limits the severity of influenza A virus infection. Mucosal Immunol. 10 (6), 1581–1593. 10.1038/mi.2017.16 28327617

[B27] Menegazzi P. Galvan M. Rotola A. Ravaioli T. Gonelli A. Cassai E. (1999). Temporal mapping of transcripts in human herpesvirus-7. J. Gen. Virol. 80 (Pt 10), 2705–2712. 10.1099/0022-1317-80-10-2705 10573164

[B28] North R. A. Surprenant A. (2000). Pharmacology of cloned P2X receptors. Annu. Rev. Pharmacol. Toxicol. 40, 563–580. 10.1146/annurev.pharmtox.40.1.563 10836147

[B29] Orioli E. De Marchi E. Giuliani A. L. Adinolfi E. (2017). P2X7 receptor orchestrates multiple signalling pathways triggering inflammation, autophagy and metabolic/trophic responses. Curr. Med. Chem. 24 (21), 2261–2275. 10.2174/0929867324666170303161659 28266268

[B30] Ou A. Gu B. J. Wiley J. S. (2018). The scavenger activity of the human P2X7 receptor differs from P2X7 pore function by insensitivity to antagonists, genetic variation and sodium concentration: relevance to inflammatory brain diseases. Biochim. Biophys. Acta Mol. Basis Dis. 1864 (4 Pt A), 1051–1059. 10.1016/j.bbadis.2018.01.012 29329985

[B31] Park J. H. Kim Y. C. (2017). P2X7 receptor antagonists: a patent review, (2010-2015). Expert Opin. Ther. Pat. 27 (3), 257–267. 10.1080/13543776.2017.1246538 27724045

[B32] Rassendren F. Buell G. N. Virginio C. Collo G. North R. A. Surprenant A. (1997). The permeabilizing ATP receptor, P2X7. Cloning and expression of a human cDNA. J. Biol. Chem. 272 (9), 5482–5486. 10.1074/jbc.272.9.5482 9038151

[B33] Reynaud J. M. Horvat B. (2013). Animal models for human herpesvirus 6 infection. Front. Microbiol. 4, 174. 10.3389/fmicb.2013.00174 23847599 PMC3701164

[B34] Rizzo R. Fuzzi B. Stignani M. Criscuoli L. Melchiorri L. Dabizzi S. (2007a). Soluble HLA-G molecules in follicular fluid: a tool for oocyte selection in IVF? J. Reprod. Immunol. 74 (1-2), 133–142. 10.1016/j.jri.2007.02.005 17399800

[B35] Rizzo R. Melchiorri L. Stignani M. Baricordi O. R. (2007b). HLA-G expression is a fundamental prerequisite to pregnancy. Hum. Immunol. 68 (4), 244–250. 10.1016/j.humimm.2006.10.012 17400059

[B36] Rizzo R. Ferrari D. Melchiorri L. Stignani M. Gulinelli S. Baricordi O. R. (2009). Extracellular ATP acting at the P2X7 receptor inhibits secretion of soluble HLA-G from human monocytes. J. Immunol. 183 (7), 4302–4311. 10.4049/jimmunol.0804265 19748989

[B37] Rizzo R. Vercammen M. van de Velde H. Horn P. A. Rebmann V. (2011). The importance of HLA-G expression in embryos, trophoblast cells, and embryonic stem cells. Cell Mol. Life Sci. 68 (3), 341–352. 10.1007/s00018-010-0578-1 21080028 PMC11114702

[B38] Rizzo R. Soffritti I. D’Accolti M. Bortolotti D. Di Luca D. Caselli E. (2017). HHV-6A/6B Infection of NK cells modulates the expression of miRNAs and transcription factors potentially associated to impaired NK activity. Front. Microbiol. 8, 2143. 10.3389/fmicb.2017.02143 29163428 PMC5671584

[B39] Rizzo R. D’Accolti M. Bortolotti D. Caccuri F. Caruso A. Di Luca D. (2018). Human Herpesvirus 6A and 6B inhibit *in vitro* angiogenesis by induction of Human Leukocyte Antigen G. Sci. Rep. 8 (1), 17683. 10.1038/s41598-018-36146-0 30523283 PMC6283866

[B40] Rizzo R. Bortolotti D. Gentili V. Rotola A. Bolzani S. Caselli E. (2019). KIR2DS2/KIR2DL2/HLA-C1 haplotype is associated with Alzheimer’s disease: implication for the role of herpesvirus infections. J. Alzheimers Dis. 67 (4), 1379–1389. 10.3233/JAD-180777 30689576

[B41] Roger S. Mei Z. Z. Baldwin J. M. Dong L. Bradley H. Baldwin S. A. (2010). Single nucleotide polymorphisms that were identified in affective mood disorders affect ATP-activated P2X7 receptor functions. J. Psychiatr. Res. 44 (6), 347–355. 10.1016/j.jpsychires.2009.10.005 19931869

[B42] Santoro F. Kennedy P. E. Locatelli G. Malnati M. S. Berger E. A. Lusso P. (1999). CD46 is a cellular receptor for human herpesvirus 6. Cell 99 (7), 817–827. 10.1016/s0092-8674(00)81678-5 10619434

[B43] Sanz J. M. Falzoni S. Rizzo R. Cipollone F. Zuliani G. Di Virgilio F. (2014). Possible protective role of the 489C > T P2X7R polymorphism in Alzheimer’s disease. Exp. Gerontol. 60, 117–119. 10.1016/j.exger.2014.10.009 25456845 PMC4266448

[B44] Savio L. E. B. Coutinho-Silva R. (2019). Immunomodulatory effects of P2X7 receptor in intracellular parasite infections. Curr. Opin. Pharmacol. 47, 53–58. 10.1016/j.coph.2019.02.005 30901737

[B45] Savio L. E. B. de Andrade Mello P. da Silva C. G. Coutinho-Silva R. (2018). The P2X7 receptor in inflammatory diseases: Angel or Demon? Front. Pharmacol. 9, 52. 10.3389/fphar.2018.00052 29467654 PMC5808178

[B46] Sluyter R. (2017). The P2X7 Receptor. Adv. Exp. Med. Biol. 1051, 17–53. 10.1007/5584_2017_59 28676924

[B47] Stokes L. Fuller S. J. Sluyter R. Skarratt K. K. Gu B. J. Wiley J. S. (2010). Two haplotypes of the P2X(7) receptor containing the Ala-348 to Thr polymorphism exhibit a gain-of-function effect and enhanced interleukin-1beta secretion. FASEB J. 24 (8), 2916–2927. 10.1096/fj.09-150862 20360457

[B48] Surprenant A. Rassendren F. Kawashima E. North R. A. Buell G. (1996). The cytolytic P2Z receptor for extracellular ATP identified as a P2X receptor (P2X7). Science 272 (5262), 735–738. 10.1126/science.272.5262.735 8614837

[B49] Tsimis M. E. Lei J. Rosenzweig J. M. Arif H. Shabi Y. Alshehri W. (2017). P2X7 receptor blockade prevents preterm birth and perinatal brain injury in a mouse model of intrauterine inflammation. Biol. Reprod. 97 (2), 230–239. 10.1093/biolre/iox081 29044426

[B50] Zandberg M. van Son W. J. Harmsen M. C. Bakker W. W. (2007). Infection of human endothelium *in vitro* by cytomegalovirus causes enhanced expression of purinergic receptors: a potential virus escape mechanism? Transplantation 84 (10), 1343–1347. 10.1097/01.tp.0000287598.25493.a5 18049120

[B51] Zhang C. Yan Y. He H. Wang L. Zhang N. Zhang J. (2019). IFN-stimulated P2Y13 protects mice from viral infection by suppressing the cAMP/EPAC1 signaling pathway. J. Mol. Cell Biol. 11 (5), 395–407. 10.1093/jmcb/mjy045 30137373 PMC7107496

