# Supplementary figures and images for: The P2X7 Receptor 489C>T Gain of Function Polymorphism Favors HHV-6A Infection and Associates With Female Idiopathic Infertility
Source: Front Pharmacol. 2020 Feb 21;11:96. doi: 10.3389/fphar.2020.00096 (PMC7046806; doi:10.3389/fphar.2020.00096)

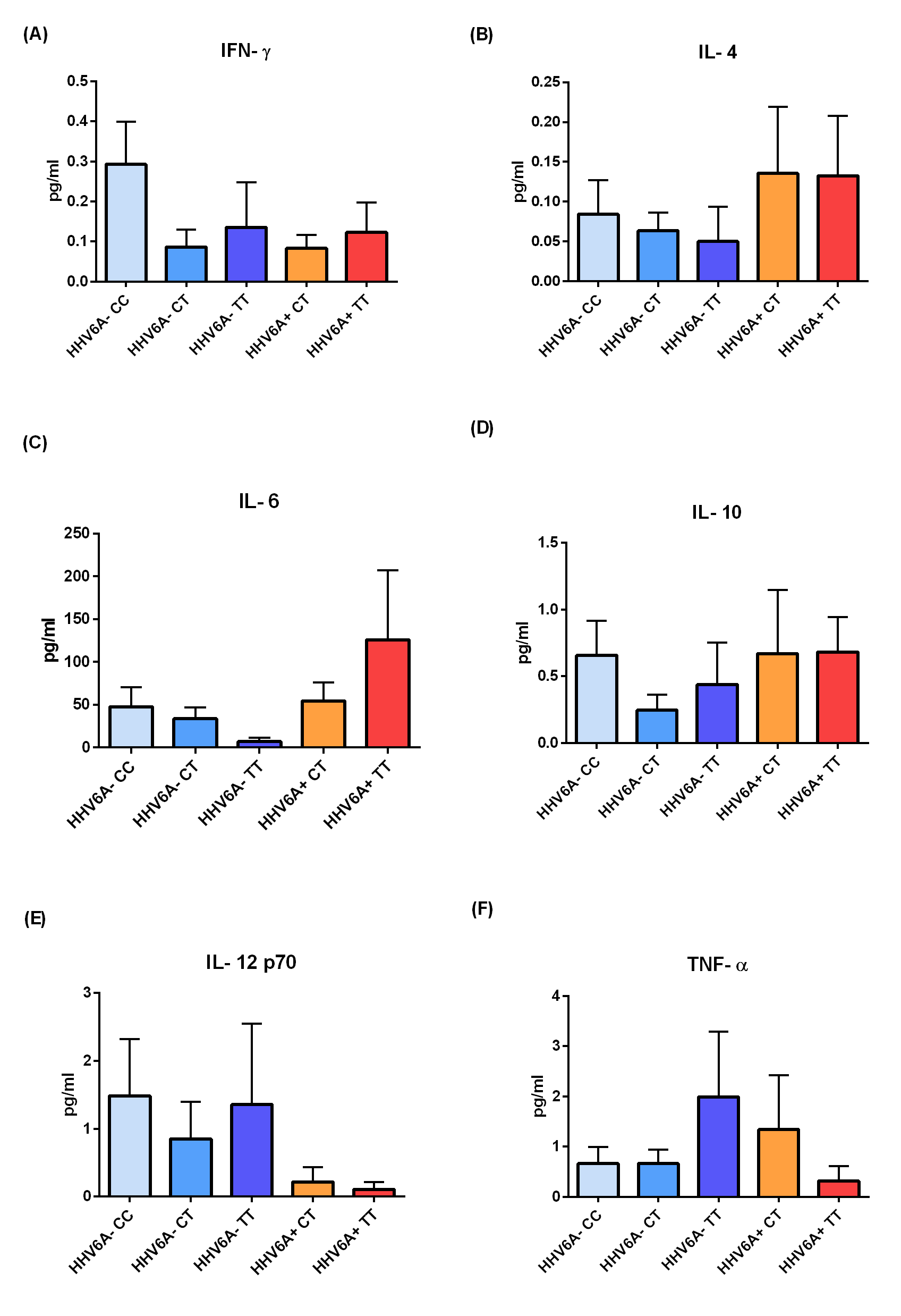

Supplement: Supplementary Figure 2 — (A) IFN-γ, (B) IL-4, (C) IL-6, (D) IL-10, (E) IL-12p70 and (F) TNF-α concentrations were evaluated in uterine flushing samples from infertile women (n = 50) and analyzed according to P2X7R 489 genotype and HHV-6A infection as follows: HHV-6A negative with P2X7R 489 genotype CC (n = 16), HHV-6A negative with P2X7R 489 genotype CT (n = 19), HHV-6A negative with P2X7R 489 genotype TT (n = 4), HHV-6A positive with P2X7R 489 genotype CT (n = 7), HHV-6A positive with P2X7R 489 genotype TT (n = 4). They did not significantly differ within the tested populations by ANOVA test (p = 0.2503, p = 0.7628, p = 0.2436, p = 0.6017, p = 0.7609, p = 0.5105, respectively). [file Image_2.tif]
